# [(*Z*)-*N*-(2-Chloro­phen­yl)-*O*-methyl­thio­carbamato-κ*S*](triphenyl­phosphine-κ*P*)gold(I)

**DOI:** 10.1107/S160053680904906X

**Published:** 2009-11-21

**Authors:** Primjira P. Tadbuppa, Edward R. T. Tiekink

**Affiliations:** a Department of Chemistry, National University of Singapore, Singapore 117543; b Department of Chemistry, University of Malaya, 50603 Kuala Lumpur, Malaysia

## Abstract

In the title compound, [Au(C_8_H_7_ClNOS)(C_18_H_15_P)], the Au^I^ atom has a near-linear geometry, defined by an *S*,*P*-donor set [S—Au—P = 175.09 (5)°]. The proximity of the meth­oxy O atom to Au may be responsible for the deviation from linearity [Au⋯O = 2.959 (4) Å].

## Related literature

For structural systematics and luminescence properties of phosphinegold(I) carbonimidothio­ates, see: Ho *et al.* (2006 ▶); Ho & Tiekink (2007 ▶); Kuan *et al.* (2008 ▶). For the synthesis, see: Hall *et al.* (1993 ▶).
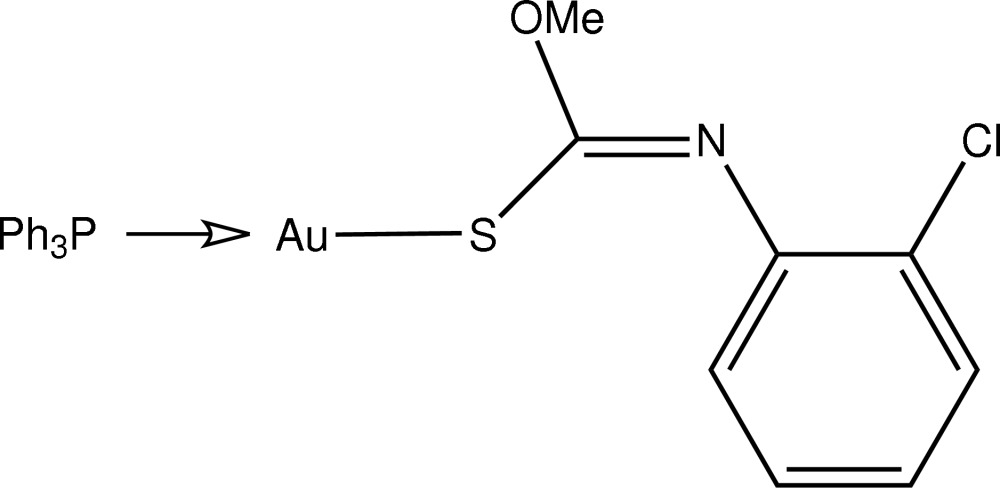


## Experimental

### 

#### Crystal data

[Au(C_8_H_7_ClNOS)(C_18_H_15_P)]
*M* *_r_* = 659.89
Monoclinic, 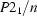

*a* = 8.9388 (5) Å
*b* = 26.2804 (15) Å
*c* = 10.3233 (6) Å
β = 96.599 (1)°
*V* = 2409.0 (2) Å^3^
*Z* = 4
Mo *K*α radiation
μ = 6.39 mm^−1^
*T* = 223 K
0.13 × 0.10 × 0.07 mm

#### Data collection

Bruker SMART CCD diffractometer
Absorption correction: multi-scan (*SADABS*; Bruker, 2000 ▶) *T*_min_ = 0.521, *T*_max_ = 1
17015 measured reflections
5537 independent reflections
4400 reflections with *I* > 2σ(*I*)
*R*_int_ = 0.047

#### Refinement

*R*[*F* ^2^ > 2σ(*F* ^2^)] = 0.032
*wR*(*F* ^2^) = 0.085
*S* = 1.04
5537 reflections
290 parameters
H-atom parameters constrained
Δρ_max_ = 1.17 e Å^−3^
Δρ_min_ = −0.54 e Å^−3^

### 

Data collection: *SMART* (Bruker, 2000 ▶); cell refinement: *SAINT* (Bruker, 2000 ▶); data reduction: *SHELXTL* (Bruker, 2000 ▶); program(s) used to solve structure: *PATTY* in *DIRDIF92* (Beurskens *et al.*, 1992 ▶); program(s) used to refine structure: *SHELXL97* (Sheldrick, 2008 ▶); molecular graphics: *DIAMOND* (Brandenburg, 2006 ▶); software used to prepare material for publication: *publCIF* (Westrip, 2009 ▶).

## Supplementary Material

Crystal structure: contains datablocks global, I. DOI: 10.1107/S160053680904906X/hb5235sup1.cif

Structure factors: contains datablocks I. DOI: 10.1107/S160053680904906X/hb5235Isup2.hkl

Additional supplementary materials:  crystallographic information; 3D view; checkCIF report

## Figures and Tables

**Table 1:** Selected bond lengths (Å)

Au—S1	2.3086 (12)
Au—P1	2.2508 (12)

**Fig. 1.**
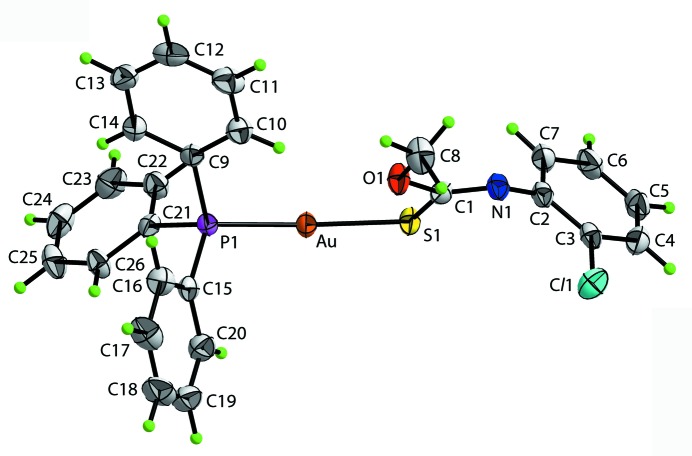
Molecular structure of (I) showing displacement ellipsoids at the 50% probability level.

## supplementary crystallographic information

### Comment

The motivation for systematic studies of phosphinegold(I) thiocarbamides, *e.g.* (*R*_3_P)PAu[SC(OR')NR''], relates to the delineation of crystal packing characteristics, *e.g.* the propensity to form aurophilic (Au···Au) interactions (Ho & Tiekink, 2007; Kuan *et al.*, 2008), as well as the examination of their luminescence characteristics (Ho *et al.* 2006). The title compound, (C_5_H_5_)_3_PAu[SC(OMe)N(C_6_H_4_Cl-o)], (I), was synthesized during the course of these studies.

The thiocarbamato anion functions as a thiolate ligand in (I), Fig. 1, as seen in the magnitudes of the C1—S1 and C1═N1 bond distances of 1.762 (5) and 1.269 (6) Å, respectively; the conformation about C1═N1 is *Z*. The central SC(O)N chromophore has small but significant twists from planarity as seen in the S1–C1–N1–C2 and O1–C1–N1–C2 torsion angles of 10.0 (7) and -173.1 (4) °, respectively. The N-bound aryl ring is twisted out of this plane: the C1–N1–C2–C3 torsion angle is -126.3 (5) °. The thiocarbamato and phosphine ligands define an approximately linear *S*, *P* donor set, Table. The deviation of the S1—Au—P1 angle [175.09 (5) °] from the ideal linear geometry is ascribed to the close approach of the O1 atom [2.959 (4) Å] to Au.

### Experimental

Compound (I) was prepared following the standard literature procedure from the reaction of Ph_3_AuCl and MeOC(S)N(H)(C_6_H_4_Cl-*o*) in the presence of base (Hall *et al.*, 1993).

### Refinement

The H atoms were geometrically placed (C—H = 0.94–0.97 Å) and refined as riding with *U_iso_*(H) = 1.2–1.5*U*_eq_(C). A rotating group model was used for the methyl group. The maximum and minimum residual electron density peaks of 1.17 and 0.54 e Å^-3^, respectively, are located 0.86 Å and 0.38 Å from the Au and H20 atoms, respectively.

### Crystal data

**Table:**

[Au(C_8_H_7_ClNOS)(C_18_H_15_P)]	*F*(000) = 1280
*M**_r_* = 659.89	*D*_x_ = 1.819 Mg m^−^^3^
Monoclinic, *P*2_1_/*n*	Mo *K*α radiation, λ = 0.71069 Å
Hall symbol: -P 2yn	Cell parameters from 3828 reflections
*a* = 8.9388 (5) Å	θ = 2.4–24.2°
*b* = 26.2804 (15) Å	µ = 6.39 mm^−^^1^
*c* = 10.3233 (6) Å	*T* = 223 K
β = 96.599 (1)°	Block, colourless
*V* = 2409.0 (2) Å^3^	0.13 × 0.10 × 0.07 mm
*Z* = 4	

### Data collection

**Table:**

Bruker SMART CCD diffractometer	5537 independent reflections
Radiation source: fine-focus sealed tube	4400 reflections with *I* > 2σ(*I*)
graphite	*R*_int_ = 0.047
ω scans	θ_max_ = 27.5°, θ_min_ = 1.6°
Absorption correction: multi-scan (*SADABS*; Bruker, 2000)	*h* = −11→10
*T*_min_ = 0.521, *T*_max_ = 1	*k* = −34→33
17015 measured reflections	*l* = −13→12

### Refinement

**Table:**

Refinement on *F*^2^	Primary atom site location: structure-invariant direct methods
Least-squares matrix: full	Secondary atom site location: difference Fourier map
*R*[*F*^2^ > 2σ(*F*^2^)] = 0.032	Hydrogen site location: inferred from neighbouring sites
*wR*(*F*^2^) = 0.085	H-atom parameters constrained
*S* = 1.04	*w* = 1/[σ^2^(*F*_o_^2^) + (0.0379*P*)^2^] where *P* = (*F*_o_^2^ + 2*F*_c_^2^)/3
5537 reflections	(Δ/σ)_max_ < 0.001
290 parameters	Δρ_max_ = 1.17 e Å^−^^3^
0 restraints	Δρ_min_ = −0.54 e Å^−^^3^

### Special details

**Table:**

Geometry. All s.u.'s (except the s.u. in the dihedral angle between two l.s. planes) are estimated using the full covariance matrix. The cell s.u.'s are taken into account individually in the estimation of s.u.'s in distances, angles and torsion angles; correlations between s.u.'s in cell parameters are only used when they are defined by crystal symmetry. An approximate (isotropic) treatment of cell s.u.'s is used for estimating s.u.'s involving l.s. planes.
Refinement. Refinement of *F*^2^ against ALL reflections. The weighted *R*-factor *wR* and goodness of fit *S* are based on *F*^2^, conventional *R*-factors *R* are based on *F*, with *F* set to zero for negative *F*^2^. The threshold expression of *F*^2^ > 2σ(*F*^2^) is used only for calculating *R*-factors(gt) *etc*. and is not relevant to the choice of reflections for refinement. *R*-factors based on *F*^2^ are statistically about twice as large as those based on *F*, and *R*- factors based on ALL data will be even larger.

### Fractional atomic coordinates and isotropic or equivalent isotropic displacement parameters (Å^2^)

**Table:**

	*x*	*y*	*z*	*U*_iso_*/*U*_eq_	
Au	0.24654 (2)	0.102793 (8)	0.096786 (18)	0.02961 (8)	
Cl1	0.89614 (16)	0.17339 (6)	−0.09989 (13)	0.0446 (3)	
S1	0.37239 (14)	0.14902 (5)	−0.04703 (12)	0.0313 (3)	
P1	0.13681 (14)	0.06089 (5)	0.25093 (12)	0.0267 (3)	
O1	0.5076 (4)	0.16834 (14)	0.1853 (3)	0.0349 (9)	
N1	0.6237 (5)	0.20523 (16)	0.0254 (4)	0.0320 (10)	
C1	0.5160 (5)	0.17848 (19)	0.0581 (5)	0.0283 (11)	
C2	0.6239 (6)	0.22061 (19)	−0.1058 (5)	0.0299 (11)	
C3	0.7475 (5)	0.21050 (19)	−0.1725 (5)	0.0301 (11)	
C4	0.7550 (6)	0.2287 (2)	−0.2976 (5)	0.0390 (13)	
H4	0.8395	0.2213	−0.3407	0.047*	
C5	0.6403 (7)	0.2573 (2)	−0.3584 (5)	0.0405 (13)	
H5	0.6450	0.2697	−0.4432	0.049*	
C6	0.5180 (6)	0.2676 (2)	−0.2937 (6)	0.0428 (14)	
H6	0.4379	0.2869	−0.3356	0.051*	
C7	0.5101 (6)	0.2503 (2)	−0.1685 (5)	0.0384 (13)	
H7	0.4265	0.2589	−0.1253	0.046*	
C8	0.6204 (7)	0.1907 (3)	0.2779 (5)	0.0468 (15)	
H8A	0.7169	0.1748	0.2702	0.070*	
H8B	0.5936	0.1855	0.3653	0.070*	
H8C	0.6271	0.2268	0.2608	0.070*	
C9	0.1331 (5)	0.10132 (18)	0.3935 (5)	0.0273 (10)	
C10	0.2482 (6)	0.1366 (2)	0.4224 (5)	0.0366 (13)	
H10	0.3225	0.1407	0.3658	0.044*	
C11	0.2530 (6)	0.1655 (2)	0.5335 (6)	0.0444 (14)	
H11	0.3326	0.1885	0.5538	0.053*	
C12	0.1430 (7)	0.1611 (2)	0.6157 (6)	0.0441 (15)	
H12	0.1479	0.1806	0.6923	0.053*	
C13	0.0248 (7)	0.1275 (2)	0.5843 (6)	0.0454 (15)	
H13	−0.0527	0.1253	0.6384	0.055*	
C14	0.0199 (6)	0.0975 (2)	0.4747 (5)	0.0379 (13)	
H14	−0.0599	0.0745	0.4547	0.045*	
C15	0.2397 (5)	0.00408 (19)	0.3076 (5)	0.0301 (11)	
C16	0.2844 (6)	−0.0052 (2)	0.4394 (5)	0.0386 (13)	
H16	0.2640	0.0185	0.5031	0.046*	
C17	0.3602 (7)	−0.0506 (2)	0.4751 (7)	0.0510 (16)	
H17	0.3904	−0.0572	0.5637	0.061*	
C18	0.3912 (7)	−0.0857 (2)	0.3836 (8)	0.0551 (18)	
H18	0.4414	−0.1161	0.4095	0.066*	
C19	0.3488 (7)	−0.0762 (2)	0.2546 (7)	0.0526 (17)	
H19	0.3709	−0.1001	0.1917	0.063*	
C20	0.2728 (6)	−0.0314 (2)	0.2154 (6)	0.0422 (14)	
H20	0.2440	−0.0253	0.1263	0.051*	
C21	−0.0550 (5)	0.03955 (19)	0.2088 (4)	0.0262 (10)	
C22	−0.1585 (6)	0.0739 (2)	0.1473 (5)	0.0367 (13)	
H22	−0.1274	0.1065	0.1245	0.044*	
C23	−0.3071 (6)	0.0596 (3)	0.1200 (6)	0.0470 (15)	
H23	−0.3778	0.0830	0.0807	0.056*	
C24	−0.3536 (6)	0.0114 (3)	0.1495 (6)	0.0462 (15)	
H24	−0.4551	0.0020	0.1294	0.055*	
C25	−0.2516 (6)	−0.0228 (2)	0.2083 (6)	0.0449 (14)	
H25	−0.2829	−0.0558	0.2283	0.054*	
C26	−0.1020 (6)	−0.0087 (2)	0.2384 (5)	0.0332 (12)	
H26	−0.0322	−0.0321	0.2791	0.040*	

### Atomic displacement parameters (Å^2^)

**Table:**

	*U*^11^	*U*^22^	*U*^33^	*U*^12^	*U*^13^	*U*^23^
Au	0.02941 (12)	0.03086 (12)	0.02991 (12)	−0.00652 (9)	0.00915 (8)	0.00018 (9)
Cl1	0.0407 (8)	0.0589 (10)	0.0340 (7)	0.0071 (7)	0.0033 (6)	−0.0004 (7)
S1	0.0317 (7)	0.0374 (8)	0.0257 (6)	−0.0082 (6)	0.0074 (5)	0.0005 (5)
P1	0.0265 (7)	0.0255 (7)	0.0290 (7)	−0.0051 (5)	0.0073 (5)	−0.0014 (5)
O1	0.035 (2)	0.045 (2)	0.0252 (19)	−0.0108 (17)	0.0040 (15)	0.0024 (16)
N1	0.030 (2)	0.032 (3)	0.035 (2)	−0.0076 (19)	0.0042 (19)	−0.0003 (19)
C1	0.026 (2)	0.032 (3)	0.028 (3)	−0.001 (2)	0.006 (2)	−0.004 (2)
C2	0.032 (3)	0.029 (3)	0.029 (3)	−0.009 (2)	0.004 (2)	−0.001 (2)
C3	0.032 (3)	0.027 (3)	0.030 (3)	−0.009 (2)	0.001 (2)	0.000 (2)
C4	0.038 (3)	0.045 (3)	0.036 (3)	−0.008 (3)	0.012 (2)	−0.004 (3)
C5	0.054 (4)	0.037 (3)	0.031 (3)	−0.006 (3)	0.008 (3)	0.008 (2)
C6	0.045 (3)	0.034 (3)	0.049 (4)	−0.001 (3)	0.003 (3)	0.013 (3)
C7	0.036 (3)	0.034 (3)	0.046 (3)	−0.003 (2)	0.010 (3)	0.008 (3)
C8	0.050 (4)	0.060 (4)	0.029 (3)	−0.018 (3)	−0.003 (3)	0.001 (3)
C9	0.026 (3)	0.025 (3)	0.031 (3)	0.002 (2)	0.004 (2)	0.002 (2)
C10	0.031 (3)	0.036 (3)	0.043 (3)	−0.003 (2)	0.003 (2)	−0.005 (2)
C11	0.040 (3)	0.039 (3)	0.052 (4)	−0.006 (3)	−0.004 (3)	−0.014 (3)
C12	0.060 (4)	0.034 (3)	0.036 (3)	0.012 (3)	−0.002 (3)	−0.006 (3)
C13	0.062 (4)	0.037 (3)	0.040 (3)	0.007 (3)	0.020 (3)	0.000 (3)
C14	0.042 (3)	0.035 (3)	0.039 (3)	−0.007 (3)	0.015 (3)	−0.003 (2)
C15	0.020 (2)	0.029 (3)	0.041 (3)	−0.006 (2)	0.006 (2)	−0.003 (2)
C16	0.033 (3)	0.040 (3)	0.042 (3)	0.000 (2)	−0.001 (2)	−0.002 (3)
C17	0.040 (3)	0.044 (4)	0.065 (4)	0.000 (3)	−0.009 (3)	0.013 (3)
C18	0.037 (4)	0.035 (3)	0.093 (6)	0.011 (3)	0.004 (3)	0.009 (4)
C19	0.043 (4)	0.041 (4)	0.078 (5)	0.004 (3)	0.023 (3)	−0.009 (3)
C20	0.037 (3)	0.041 (3)	0.050 (4)	−0.001 (3)	0.010 (3)	−0.006 (3)
C21	0.022 (2)	0.033 (3)	0.024 (2)	−0.003 (2)	0.0043 (19)	−0.004 (2)
C22	0.037 (3)	0.033 (3)	0.039 (3)	0.000 (2)	0.003 (2)	0.005 (2)
C23	0.033 (3)	0.058 (4)	0.050 (4)	0.009 (3)	0.001 (3)	0.004 (3)
C24	0.024 (3)	0.071 (5)	0.043 (3)	−0.007 (3)	0.001 (2)	−0.009 (3)
C25	0.044 (3)	0.043 (3)	0.047 (4)	−0.020 (3)	0.001 (3)	0.000 (3)
C26	0.028 (3)	0.031 (3)	0.039 (3)	−0.005 (2)	−0.004 (2)	0.006 (2)

### Geometric parameters (Å, °)

**Table:**

Au—S1	2.3086 (12)	C11—H11	0.9400
Au—P1	2.2508 (12)	C12—C13	1.386 (8)
Cl1—C3	1.746 (5)	C12—H12	0.9400
S1—C1	1.762 (5)	C13—C14	1.376 (8)
P1—C21	1.809 (5)	C13—H13	0.9400
P1—C15	1.815 (5)	C14—H14	0.9400
P1—C9	1.819 (5)	C15—C20	1.389 (7)
O1—C1	1.350 (6)	C15—C16	1.395 (7)
O1—C8	1.433 (6)	C16—C17	1.400 (8)
N1—C1	1.269 (6)	C16—H16	0.9400
N1—C2	1.414 (6)	C17—C18	1.371 (9)
C2—C7	1.383 (7)	C17—H17	0.9400
C2—C3	1.393 (7)	C18—C19	1.365 (9)
C3—C4	1.385 (7)	C18—H18	0.9400
C4—C5	1.365 (8)	C19—C20	1.396 (8)
C4—H4	0.9400	C19—H19	0.9400
C5—C6	1.372 (8)	C20—H20	0.9400
C5—H5	0.9400	C21—C26	1.380 (7)
C6—C7	1.380 (7)	C21—C22	1.393 (7)
C6—H6	0.9400	C22—C23	1.378 (8)
C7—H7	0.9400	C22—H22	0.9400
C8—H8A	0.9700	C23—C24	1.378 (9)
C8—H8B	0.9700	C23—H23	0.9400
C8—H8C	0.9700	C24—C25	1.372 (8)
C9—C14	1.390 (7)	C24—H24	0.9400
C9—C10	1.391 (7)	C25—C26	1.388 (7)
C10—C11	1.372 (7)	C25—H25	0.9400
C10—H10	0.9400	C26—H26	0.9400
C11—C12	1.376 (8)		
			
P1—Au—S1	175.09 (5)	C11—C12—C13	119.3 (5)
C1—S1—Au	102.08 (17)	C11—C12—H12	120.4
C21—P1—C15	104.7 (2)	C13—C12—H12	120.4
C21—P1—C9	105.7 (2)	C14—C13—C12	120.5 (5)
C15—P1—C9	106.0 (2)	C14—C13—H13	119.7
C21—P1—Au	117.37 (16)	C12—C13—H13	119.7
C15—P1—Au	112.61 (16)	C13—C14—C9	119.9 (5)
C9—P1—Au	109.65 (16)	C13—C14—H14	120.1
C1—O1—C8	117.1 (4)	C9—C14—H14	120.1
C1—N1—C2	119.9 (4)	C20—C15—C16	119.4 (5)
N1—C1—O1	119.8 (4)	C20—C15—P1	118.1 (4)
N1—C1—S1	126.9 (4)	C16—C15—P1	122.5 (4)
O1—C1—S1	113.2 (3)	C15—C16—C17	118.8 (5)
C7—C2—C3	117.3 (5)	C15—C16—H16	120.6
C7—C2—N1	121.8 (5)	C17—C16—H16	120.6
C3—C2—N1	120.5 (5)	C18—C17—C16	121.4 (6)
C4—C3—C2	121.4 (5)	C18—C17—H17	119.3
C4—C3—Cl1	118.5 (4)	C16—C17—H17	119.3
C2—C3—Cl1	120.1 (4)	C19—C18—C17	119.6 (6)
C5—C4—C3	120.3 (5)	C19—C18—H18	120.2
C5—C4—H4	119.9	C17—C18—H18	120.2
C3—C4—H4	119.9	C18—C19—C20	120.6 (6)
C4—C5—C6	119.0 (5)	C18—C19—H19	119.7
C4—C5—H5	120.5	C20—C19—H19	119.7
C6—C5—H5	120.5	C15—C20—C19	120.2 (6)
C5—C6—C7	121.3 (5)	C15—C20—H20	119.9
C5—C6—H6	119.3	C19—C20—H20	119.9
C7—C6—H6	119.3	C26—C21—C22	119.6 (5)
C6—C7—C2	120.7 (5)	C26—C21—P1	122.4 (4)
C6—C7—H7	119.7	C22—C21—P1	118.1 (4)
C2—C7—H7	119.7	C23—C22—C21	119.4 (5)
O1—C8—H8A	109.5	C23—C22—H22	120.3
O1—C8—H8B	109.5	C21—C22—H22	120.3
H8A—C8—H8B	109.5	C22—C23—C24	120.9 (6)
O1—C8—H8C	109.5	C22—C23—H23	119.5
H8A—C8—H8C	109.5	C24—C23—H23	119.5
H8B—C8—H8C	109.5	C25—C24—C23	119.9 (5)
C14—C9—C10	119.4 (5)	C25—C24—H24	120.1
C14—C9—P1	121.9 (4)	C23—C24—H24	120.1
C10—C9—P1	118.7 (4)	C24—C25—C26	119.9 (5)
C11—C10—C9	119.9 (5)	C24—C25—H25	120.1
C11—C10—H10	120.1	C26—C25—H25	120.1
C9—C10—H10	120.1	C21—C26—C25	120.4 (5)
C10—C11—C12	120.9 (5)	C21—C26—H26	119.8
C10—C11—H11	119.5	C25—C26—H26	119.8
C12—C11—H11	119.5		
			
C2—N1—C1—O1	−173.1 (4)	C10—C9—C14—C13	−1.8 (8)
C2—N1—C1—S1	10.0 (7)	P1—C9—C14—C13	177.5 (4)
C8—O1—C1—N1	2.5 (7)	C21—P1—C15—C20	74.9 (4)
C8—O1—C1—S1	179.8 (4)	C9—P1—C15—C20	−173.6 (4)
Au—S1—C1—N1	174.2 (4)	Au—P1—C15—C20	−53.7 (4)
Au—S1—C1—O1	−2.9 (4)	C21—P1—C15—C16	−104.3 (4)
C1—N1—C2—C7	60.3 (7)	C9—P1—C15—C16	7.2 (5)
C1—N1—C2—C3	−126.3 (5)	Au—P1—C15—C16	127.1 (4)
C7—C2—C3—C4	−1.1 (7)	C20—C15—C16—C17	−0.7 (8)
N1—C2—C3—C4	−174.9 (5)	P1—C15—C16—C17	178.4 (4)
C7—C2—C3—Cl1	179.8 (4)	C15—C16—C17—C18	0.1 (9)
N1—C2—C3—Cl1	6.1 (7)	C16—C17—C18—C19	0.6 (10)
C2—C3—C4—C5	0.1 (8)	C17—C18—C19—C20	−0.7 (10)
Cl1—C3—C4—C5	179.2 (4)	C16—C15—C20—C19	0.6 (8)
C3—C4—C5—C6	0.0 (9)	P1—C15—C20—C19	−178.6 (4)
C4—C5—C6—C7	1.0 (9)	C18—C19—C20—C15	0.1 (9)
C5—C6—C7—C2	−2.0 (9)	C15—P1—C21—C26	9.0 (5)
C3—C2—C7—C6	2.1 (8)	C9—P1—C21—C26	−102.7 (4)
N1—C2—C7—C6	175.7 (5)	Au—P1—C21—C26	134.7 (4)
C21—P1—C9—C14	22.3 (5)	C15—P1—C21—C22	−172.6 (4)
C15—P1—C9—C14	−88.5 (5)	C9—P1—C21—C22	75.7 (4)
Au—P1—C9—C14	149.7 (4)	Au—P1—C21—C22	−46.9 (4)
C21—P1—C9—C10	−158.4 (4)	C26—C21—C22—C23	1.7 (8)
C15—P1—C9—C10	90.8 (4)	P1—C21—C22—C23	−176.7 (4)
Au—P1—C9—C10	−31.0 (5)	C21—C22—C23—C24	−1.8 (9)
C14—C9—C10—C11	3.2 (8)	C22—C23—C24—C25	0.8 (9)
P1—C9—C10—C11	−176.1 (4)	C23—C24—C25—C26	0.3 (9)
C9—C10—C11—C12	−1.8 (9)	C22—C21—C26—C25	−0.6 (8)
C10—C11—C12—C13	−0.9 (9)	P1—C21—C26—C25	177.7 (4)
C11—C12—C13—C14	2.3 (9)	C24—C25—C26—C21	−0.3 (8)
C12—C13—C14—C9	−0.9 (9)		

## References

[bb1] Beurskens, P. T., Admiraal, G., Beurskens, G., Bosman, W. P., Garcia-Granda, S., Gould, R. O., Smits, J. M. M. & Smykalla, C. (1992). *The *DIRDIF* Program System*. Technical Report. Crystallography Laboratory, University of Nijmegen, The Netherlands.

[bb2] Brandenburg, K. (2006). *DIAMOND*. Crystal Impact GbR, Bonn, Germany.

[bb3] Bruker (2000). *SMART*, *SAINT* and *SADABS*. Bruker AXS Inc., Madison, Wisconsin, USA.

[bb4] Hall, V. J., Siasios, G. & Tiekink, E. R. T. (1993). *Aust. J. Chem.* **46**, 561–570.

[bb5] Ho, S. Y., Cheng, E. C.-C., Tiekink, E. R. T. & Yam, V. W.-W. (2006). *Inorg. Chem.* **45**, 8165–8174. 10.1021/ic0608243 16999414

[bb6] Ho, S. Y. & Tiekink, E. R. T. (2007). *CrystEngComm*, **9**, 368–378.

[bb7] Kuan, F. S., Ho, S. Y., Tadbuppa, P. P. & Tiekink, E. R. T. (2008). *CrystEngComm*, **10**, 548–564.

[bb8] Sheldrick, G. M. (2008). *Acta Cryst.* A**64**, 112–122. 10.1107/S0108767307043930 18156677

[bb9] Westrip, S. P. (2009). *publCIF*. In preparation.

